# Supplementary material for: A Predictive Model of Antibody Binding in the Presence of IgG-Interacting Bacterial Surface Proteins
Source: Front Immunol. 2021 Mar 22;12:629103. doi: 10.3389/fimmu.2021.629103 (PMC8019711; doi:10.3389/fimmu.2021.629103)
Supplement: Supplementary file 1 [file Image_1.pdf]

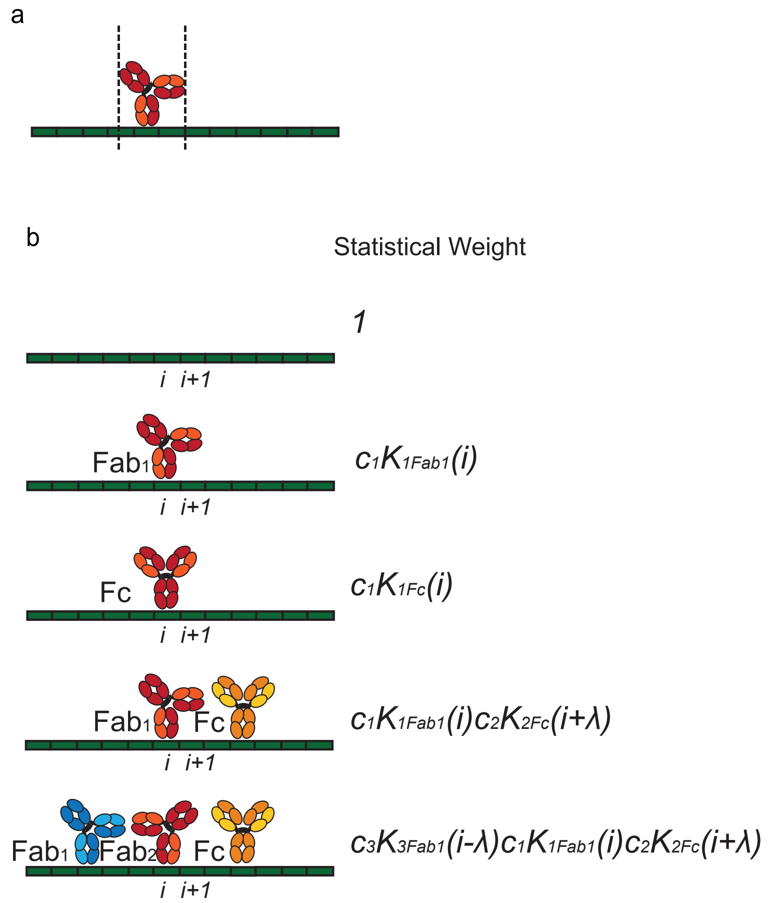

Fig. S1. a. A schematic of antibody s bound to a site on the linear bacterial protein while covering two additional sites, thus illustrating the case $\lambda = 3$ b. Examples of statistical weights. Different clonotypes are shown in different colours. The state in which no antibodies are bound to the bacterial protein has the statistical weight 1, whereas the statistical weight for a bound antibody depends on its present concentration c_s and the site and fragment specific binding constant $K_{Fab/Fc}(i)$
