# Supplementary material for: A Predictive Model of Antibody Binding in the Presence of IgG-Interacting Bacterial Surface Proteins
Source: Front Immunol. 2021 Mar 22;12:629103. doi: 10.3389/fimmu.2021.629103 (PMC8019711; doi:10.3389/fimmu.2021.629103)
Supplement: Supplementary file 2 [file Image_2.pdf]

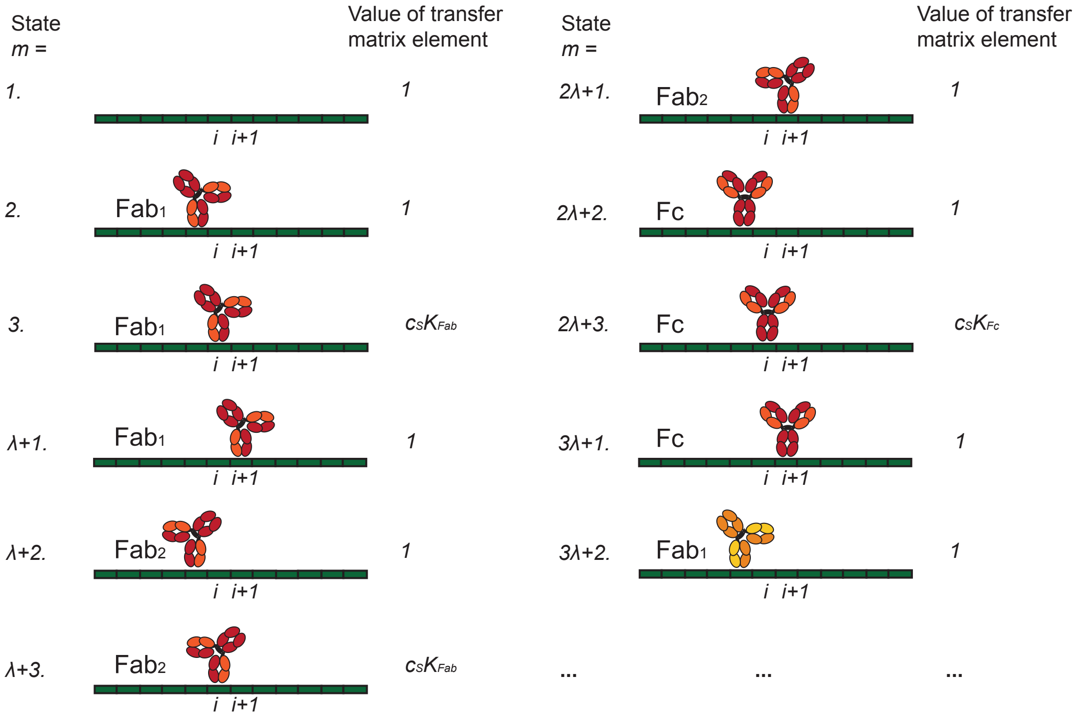

Fig. S2. The choice of enumeration adopted for our transfer matrices. The binding states m are illustrated together with their transfer matrix values for site i. All states for the first antibody clone $s = 1$ (in red) are shown, as well as the first state for the antibody $s = 2$ (in yellow). The states of the subsequent antibody clones follow the same pattern.
