# Supplementary material for: A Predictive Model of Antibody Binding in the Presence of IgG-Interacting Bacterial Surface Proteins
Source: Front Immunol. 2021 Mar 22;12:629103. doi: 10.3389/fimmu.2021.629103 (PMC8019711; doi:10.3389/fimmu.2021.629103)
Supplement: Supplementary file 3 [file Image_3.pdf]

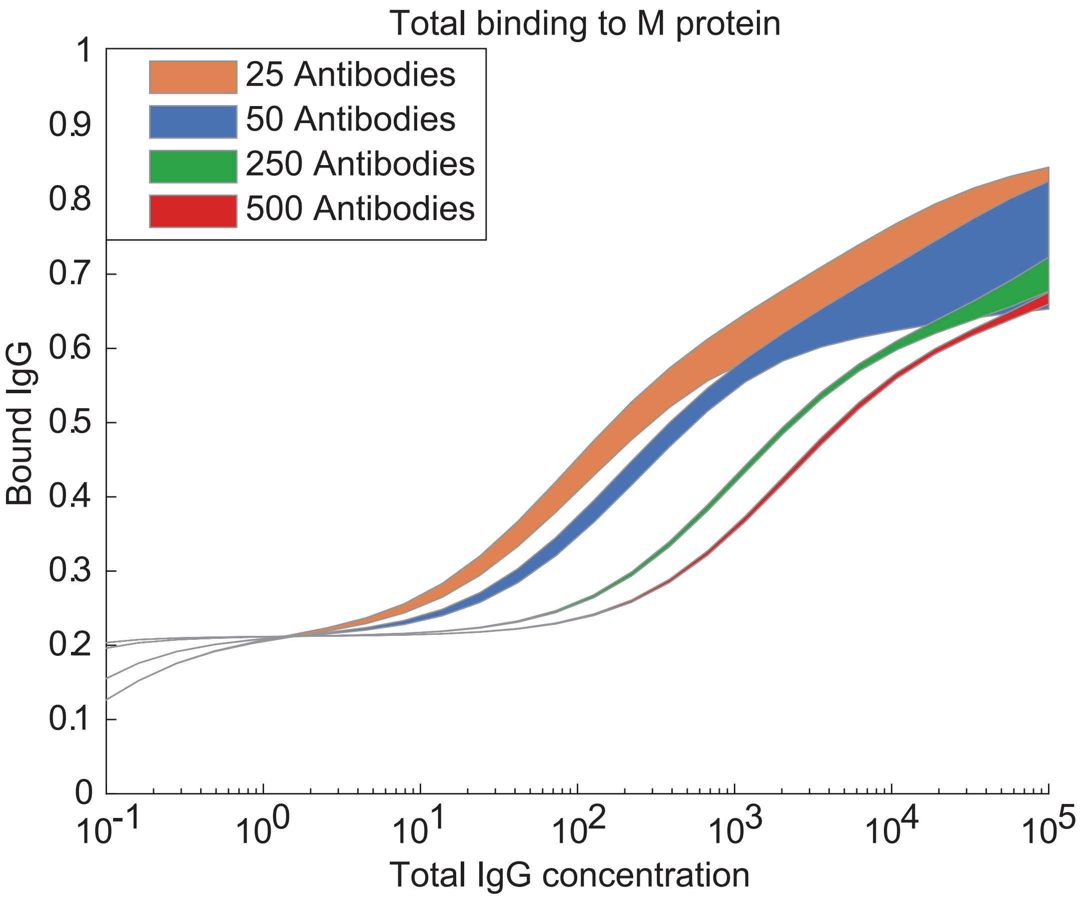

Fig. S3. The standard deviation calculated from 10 computed curves of total binding to M protein, each with a different set of randomly generated epitopes, for polyclonal IgG samples with different numbers antibody clones S. The simulation indicates that computations with the model are more stable for an increasing S.
