# Supplementary material for: A Predictive Model of Antibody Binding in the Presence of IgG-Interacting Bacterial Surface Proteins
Source: Front Immunol. 2021 Mar 22;12:629103. doi: 10.3389/fimmu.2021.629103 (PMC8019711; doi:10.3389/fimmu.2021.629103)
Supplement: Supplementary file 4 [file Image_4.pdf]

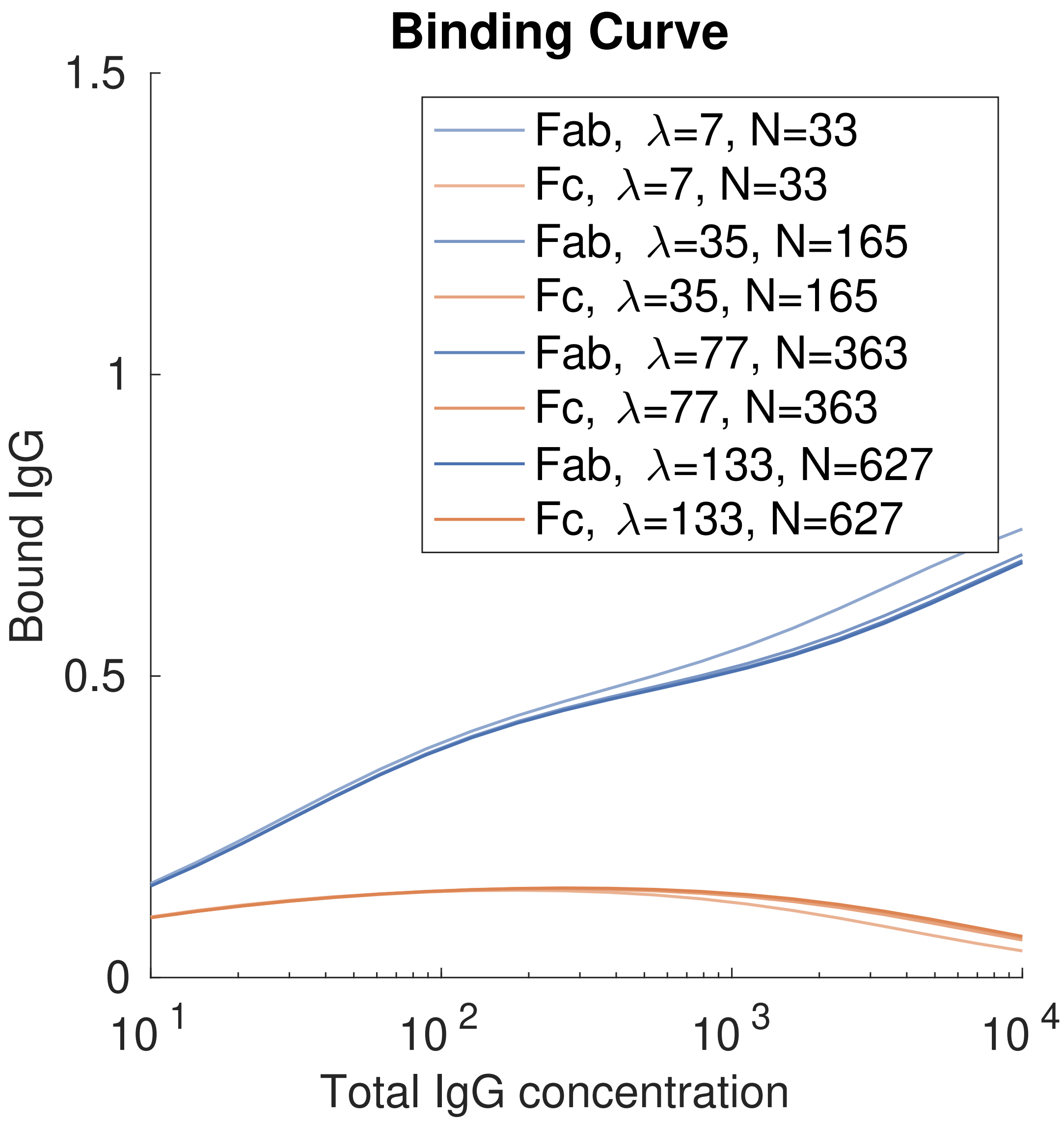

Fig. S4. Fab and Fc binding curves calculated for a polyclonal IgG sample with values set as for pooled IgG. N and λ are increased proportionally from the set values 33 and 7 respectively, as described in the methods section.
