# Supplementary material for: A Predictive Model of Antibody Binding in the Presence of IgG-Interacting Bacterial Surface Proteins
Source: Front Immunol. 2021 Mar 22;12:629103. doi: 10.3389/fimmu.2021.629103 (PMC8019711; doi:10.3389/fimmu.2021.629103)
Supplement: Supplementary file 5 [file Image_5.pdf]

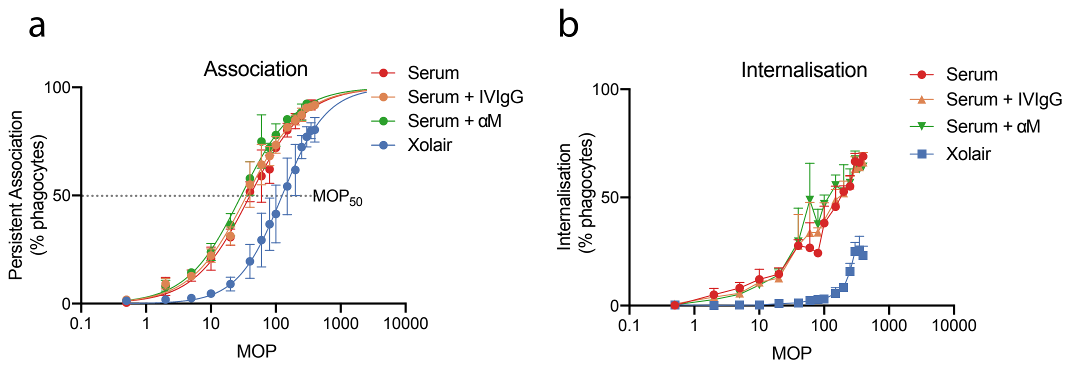

Fig. S5. Phagocytosis experiments The figure shows how added monoclonal or pooled IgG affects phagocytosis of *S. pyogenes* in serum. $20\,\mu g/ml$ of either αM or IVIgG is added to 5% of serum from donor 4. $500\,\mu g/ml$ of Xolair is used here as a baseline for non-Fc-mediated association. These are the full curves used for the data shown in Figure 5. **a.** Association of phagocytes and bacteria treated with different antibody samples. These curves depict the percentage of present phagocytes that have associated with at least one bacteria as a function of the multiplicity of prey (MOP). **b.** The internalisation curves depict the percentage of phagocytes that have internalised one or more bacteria as a function of MOP. The data shown is from three experimental repeats and the error bars indicate standard deviation ($n = 3$, mean $\pm$ SD).
